# Translation, transcultural adaptation, reliability and validation of the pelvic organ prolapse quality of life (P-QoL) in Amharic

**DOI:** 10.1186/s12955-019-1079-z

**Published:** 2019-01-14

**Authors:** Tadesse Belayneh, Abebaw Gebeyehu, Mulat Adefris, Guri Rortveit, Tinsae Genet

**Affiliations:** 1 Institute of Public Health, College of Medicine and Health Sciences, University of Gondar, P. O. Box –196, Gondar, Ethiopia; 2 Department of Obstetrics and Gynecology, School of Medicine, University of Gondar, Gondar, Ethiopia; 3 Research Group for General Practice, Department of Global Public Health and Primary Care, University of Bergen, Bergen, Norway; 4 Research Unit for General Practice, Uni Research Health, Bergen, Norway

**Keywords:** Pelvic organ prolapse, Health related quality of life, Amhara, Ethiopia

## Abstract

**Background:**

The Prolapse Quality of Life (P-QoL) is a disease-specific instrument designed to measure the health-related quality of life in women with prolapse; however, there is no Amharic version of the instrument. The aim of this study were to translate the P-QoL into Amharic and evaluate its psychometric properties among adult women.

**Methods:**

We followed an intercultural adaptation procedure to translate and adapt the P-QoL. A forward–backward translation, face validity interviews with experts and cognitive debriefing of the translated version with ten adults from the target group were performed. The Amharic version was then completed by 230 adult women with and without POP symptoms. All women were examined using a simplified Pelvic Organ Prolapse Quantification (SPOP-Q) system. We examined internal consistency (Cronbach’s alpha) and test–retest reliability (intraclass correlation coefficient = ICC). Confirmatory factor analysis (CFA) was conducted and model fit was discussed. We extracted a new factor structure by exploratory factor analysis (EFA). Criterion validity was also assessed against the SPOP-Q stage.

**Results:**

The translated measure was found acceptable by the experts and target group, with only minor adaptations required for the Amharic context. It had high internal consistency (*α* = 0.96) and test–retest reliability (ICC = 0.87; *p* <  0.001). In CFA results, the model fit indices were unacceptable (CFI = 0.69, RMSEA = 0.17, SRMR = 0.43, TLI = 0.65, and PCLOSE = 0.00). EFA extracted three-factor with satisfactory convergent and discriminant validity. The P-QoL median scores were significantly higher in symptomatic women (Mann-Whitney U Test; *p* <  0.001). The score was also significantly correlated with stage of prolapse (Spearman’s correlation coefficient = 0.42 to 0.64, *p* <  0.001).

**Conclusions:**

The P-QoL scale was successfully translated to Amharic and appears feasible, reliable and valid for Amharic-speaking women. Factor analysis confirmed a three-factor structure, inconsistent with the original English version. Further studies are needed to evaluate responsiveness of the Amharic P-QoL score.

**Electronic supplementary material:**

The online version of this article (10.1186/s12955-019-1079-z) contains supplementary material, which is available to authorized users.

## Background

Pelvic organ prolapse (POP) is the downward decent of the female pelvic organs (vagina, uterus, bladder, and/or rectum) into or through the vagina [1]. Globally around 20 to 50% of women suffer from POP [2, 3] and risk increases with age, parity and heavy lifting [2, 4].

POP exhibit multiple symptoms [5] that negatively impact women’s quality of life [6]. Health-related quality of life (HRQOL) is a composite health outcome [7] implying several subsets of function: physical, psychosocial and sexual [2, 8]. In low-income countries, women are exposed to known and probable risk factors such as high fertility rate, early childbirth, little access to treatment, limited and poor obstetric care, considerable physical burdens and finally, socio-cultural beliefs that hinder to seeking medical advice [4, 9, 10]. As a result POP might be more common, generally more severe and disproportionally affect women’s life in low-income [2, 9] compared to high-income countries. In Ethiopia, gynecological problems are important health problems affecting maternal health outcomes [11] and studies have shown that 9.4 to 55.1% of women suffer from POP [4, 12]. The country has almost all the risks favorable for POP [4, 9, 10] and POP accounted for ~ 41% of major gynecological operations in clinical setups [13].

Severity and impact of POP symptoms on HRQoL are important outcome measure in the management of POP as it reveal important aspects of the patient’s subjective experience [6, 14]. To this end, measuring HRQoL requires the use of a valid and reliable questionnaire. This enables comparison of outcome measures and thereby increases the accuracy of measurement [15]. Given the growing use of HRQoL as a surrogate outcome measure, there are considerable work in the development, adaptation and translation of condition-specific instrument in the field of Urogynecology [6]. For instance, a review by Al-Badr [16], identified four instruments specifically designed or adapted to evaluate HRQoL in women with POP: the Pelvic Floor Distress Inventory (PFDI), the Pelvic Floor Impact Questionnaire (PFIQ) [17], the Prolapse Quality of Life (P-QoL) [18] and the electronic Personal Assessment Questionnaire Pelvic Floor (ePAQ-PF) [19].

The P-QoL, originally written in English, was developed in 2005 to measure severity of symptom and its impact on the HRQoL [18]. The questionnaire contains 20 items that were grouped in 9 domains. The grouping of questions in each domain was chosen because the questions related to a particular aspect of HRQoL [18]. It has been translated to and validated in several language including Turkish [20], Portuguese [21], German [22], Dutch [23], Thai [24], Slovakian [25], Brazilian [26], Persian [27], Spanish [28] and Afrikaans [29]. The initial validation and subsequent cross-validations have assessed psychometric properties (reliability, validity and responsiveness) and both reported as a reliable and valid instrument [18, 20–29]. However, neither of the studies performed factor analysis to identify or confirm the *aforementioned* domains. Even in the absence of factor analysis, a 9-factor structure was reported in the translated studies [20–23, 25–28].

Instrument measuring health status needs evaluation of psychometric properties [30, 31] as the performance of an instrument may differ between populations and in various cultures [30]. Thus, the evaluation of any measure should be conducted within the population and setting in which it is going to be used. To our knowledge the psychometric properties of the P-QoL have not been evaluated in Amharic. Amharic, the official working language in Ethiopia, is spoken as the first language in the region where the study was conducted [32]. The lack of a validated Amharic questionnaire investigating HRQoL in patients experiencing POP limits studies and effective outcome measurement in Amharic-speaking patients in Ethiopia. Therefore, this study aimed to translate and adapt the P-QoL into Amharic and test its psychometric properties (internal consistency, test–retest reliability, content, construct, and criterion validity).

## Methods

This cross-sectional study was conducted in two phases. In phase I, translation and adaptation of the P-QoL from English into Amharic were undertaken. In phase II, psychometric validation of the Amharic version was performed.

### The original P-QoL questionnaire

P-QoL is a specific, multidimensional questionnaire with 20 items/questions. All questions, except the first which has five points, are assessed on a four-point scoring system (0 = none/never, 1 = slightly/sometimes, 2 = moderately/often, 3 = a lot/all the time). This scoring system is not a Likert scale, which is based on the fact that the intervals between two levels are all equal, i.e. the difference between ‘slightly’ and ‘moderately’ (equal to one point) may not be the same as that between ‘moderately’ and ‘a lot’ (also equal to one point). As such, it is incorrect to compute as ordinal scale to determine subscale scores. Thus, we considered items as continuous. The items were attributed to 9 domains that were transformed into a scale of 0 = (better HRQoL) excellent to 100 = (impaired HRQoL) poor: General Health Perception (GHP, one item: 1), Prolapse Impact (PI, one item: 2), Role Limitation (RL, two items: 3–4), Physical Limitation (PL, two items: 5–6), Social Limitation (SL, two items: 7–8), Personal Relationships (PR, three items: 9–11), Emotions (E, three items: 12–14), Sleep/Energy (SE, two items: 15–16), and Severity Measurement (SM, four items:17–20).

### Phase 1: Translation and adaptation of P-QoL into Amharic

After obtaining permission from the developers, we followed a standard procedure in five stages, according to the established guidelines for translation and adaptation: (1) forward translation, (2) synthesis of translations, (3) back-translation, (4) consolidation of translations by a committee of experts and (5) pre-test [33–35].

#### Stage 1- forward translation

Translation was performed by three (gynecologist, reproductive health officer and English instructor) independent native Amharic speakers fluent in English.

#### Stage 2- synthesis of the translations

A common Amharic version was created using the three translated versions through consensus between the authors and two other bilingual experts.

#### Stage 3- Back translation

The synthesis version created at the second stage was used for back- translation process. Three translators (different to stage 1) fluent in English and Amharic conducted the back-translations independently. Both were blinded and naïve to the English and translated version. The original and back-translated versions were checked for discrepancies by the authors and then referred back to the developers for conceptual and semantic equivalence. Changes, if any, were incorporated and the *first Amharic version* was produced.

#### Stage 4- expert committee review

An expert committee (*n* = 7) with medical, public health, allied health science, and sociology backgrounds subsequently reviewed the final forward and backward-translations. Consultations were conducted in person and the principal author (TB) coordinated this stage. Semantic, idiomatic, experiential and conceptual equivalence of the translated version were evaluated. Any issues raised were addressed, and a preliminary version was created and circulated among review members. Moreover, expert committee were asked to evaluate the suitability of each item and rate its relevance. Agreement was then calculated using Content Validity Index (CVI) [36]. Subsequently a *second Amharic version* was produced.

#### Stage 5- pre-test

To evaluate the equivalence and comprehensibility of the translated version, the second Amharic version was face-validated and pretested. Ten women who had stage 3/4, aged 41–60 years, speak and understand Amharic were included. An in-depth interview was conducted with each participant by an experienced female sociologist after completing the Amharic P-QoL. The interview aimed to identify the participants’ opinion on the questionnaire’s usability, applicability, and completeness. In addition, they were asked about the difficulties in understanding the items and instructions, the misunderstanding of words, the clarity of the response options and/or whether the questionnaire missed any aspects of HRQoL. Data were then discussed in the research team and decisions were made whether changes in the questionnaire were necessary. The interviews were conducted among women admitted to a gynecology ward at the University of Gondar Hospital where the psychometric testing was planned. The length of the interview was on average 30 min, including questionnaire completion.

### Phase 2: Psychometric validation of the Amharic P-QoL questionnaire

#### Study participants

All women aged ≥18 years, with or without POP symptoms, willing to participate in the studyand who visit the Gynecology Outpatient Clinic of the University of Gondar Hospital between December 2017 and March 2018 were eligible for inclusion. However, women who had a psychiatric problem, could not speak or understand Amharic, had undergone previous POP surgery, had a known or suspected pregnancy, were postpartum (first 6 weeks following childbirth), had palpable pelvic mass (uterine, ovarian, colorectal, bladder) or had history of acute symptoms of urinary tract infection were excluded from the study.

Study participants were identified by one of the research team (MA) before undergone symptom screening and pelvic examination. Symptoms of POP were assessed (MA) using two questions [4, 37]: Do you have a feeling of bulging/pressure or something coming down through the vagina? Do you have a visible mass protruding from the vagina? If the participant had experienced one or both of these problems in the past 1 year, they were considered to have symptoms of POP and were defined as symptomatic.

#### Sample size

Sample size was determined based on the recommendations of at least 5 to 10 subjects per item of the instrument by the Consensus-based Standards for the Selection of Health Measurement Instruments (COSMIN) [30]. To this end, the minimum estimated sample was 200. But we included 30 participants to protect against dropout and missing responses. Then the final participants were 230.

#### Data collection

Patients were recruited consecutively and a two-stage strategy was used to collect data. First, a face-to-face interview was conducted by two female Midwifery Nurses using the translated P-QoL at the outpatient visit (baseline data). These data collectors were not involved in pre-testing. After completing the questionnaire, all women were asked to volunteer for a pelvic examination. One research team member (TG) blinded to the questionnaire score performed the pelvic examination. The simplified Pelvic Organ Prolapse Quantification (S-POPQ) staging system was applied [38]. Pelvic examination was supervised by the research team gynaecologist (MA). Pelvic examination was done after the woman emptied her bladder. After receiving an explanation of the procedure, the participant was requested to lie on an examination couch in the lithotomy position. A disarticulated Graves speculum was inserted into the vagina. The posterior vaginal wall was retracted to observe the descent of the anterior vaginal wall and the degree of protrusion in relation to hymenal ring with strain or cough. Secondly, the anterior vaginal wall was retracted to observe a descent of the posterior vaginal wall during straining. In accordance with the method, no measuring device was used. The examiner estimated the degree of descent by observing the points on the anterior and posterior vaginal segments that were used to represent the respective walls. The point descent in relation to the hymenal ring while performing Valsalva or cough was recorded as the stage in the three areas examined (anterior, posterior and apical/cervix) and the final stage was the maximum one from the three measurements. Accordingly, women were assigned a SPOPQ stage as: stage 0, no prolapse; stage 1, leading point of the wall of the vagina or cervix remains at least 1 cm above the hymenal ring; stage 2, leading point descends to the introitus, defined as an area extending from 1 cm above to 1 cm below the hymenal ring; stage 3, leading point descends > 1 cm outside the hymenal ring, but does not form a complete vaginal vault eversion or procidentia uteri, and stage 4, complete vaginal vault eversion or procidentia uteri [38].

To measure the test–retest reliability, a randomly selected patients (*n* = 70) were asked to complete the questionnaire 2 weeks later. Patients were selected at random for to maximize the probability that the patients who received the questionnaire were representative of the sample population. The follow-up assessment was performed with face-to-face interviews by same data collectors who collected the baseline. Stability was evaluated by Patient Global Impression of Change (PGIC) scale [39] using the above data collectors. The PGIC evaluates overall health status as perceived by the patient in a seven-point single-item scale ranging from ‘very much worse’ to ‘very much improved’. For descriptive purposes, patients were classified into three categories according to the PGIC score: disease deterioration (very much worse, much worse and minimally worse), stable disease (no change) or disease improvement (very much improved, much improved and minimally improved) since the initial baseline visit. Women were considered stable if she rate “no change” on the PGIC scale [40]. The PGIC have been implemented and/or validated in clinical studies of patients with urogenital prolapse [41]. The questionnaire was translated from English to Amharic without back-translation before use. In this study women were considered stable if she scored “no change or almost the same” on the scale.

#### Statistical analysis

Sociodemographic characteristics and selected clinical background information were described with descriptive statistics. The responses were checked for completeness and partly completed questionnaires were removed prior to analysis. When necessary, items were recoded and transformed [18]. Semantic, idiomatic, experiential and conceptual equivalences were evaluated using content and face validity and acceptability. However, measurement equivalence was evaluated with test-retest reliability, internal consistency, and construct and criterion validity based on the COSMIN recommendations [42]. The significance level was set as 0.05.

**Content validity**, whether all domains of the P-QoL would cover all the appropriate domains of HRQoL, was evaluated. Questionnaires that demonstrate content validity should have few missing responses, use the full range of scores with little skew, and have few ceiling (best possible score) or floor (poorest possible score) effects. **Face validity**, the extent to which a questionnaire is a logical measure of what it intends to measure in the opinion of the experts and patients [43], was evaluated by the expert committee throughout the adaptation process and the pre-test through qualitative analysis of the comments provided. The experts were asked to make remarks or comments on the plausibility of the questions, the comprehensiveness, and the relevance of a scale ranging from 1 to 4 (very relevant to irrelevant). Expert agreement on relevance was calculated using the CVI, and agreement ≥80% was considered acceptable [36]. Moreover, **acceptability**, the extent to which an instrument is acceptable to participants, was evaluated using the estimated time required to fill out the questionnaire, percentage of fully completed questionnaire, percentage of difficult/distressing item, and levels of missing data [44].

**Reliability** was assessed using agreement and consistency indices. Cronbach’s alpha was computed to assess the internal consistency of subscale and items in the P-QoL questionnaire, and values of ≥0.7 were considered adequate [30]. We further analyzed item-to-subscale and item-to-total correlations to evaluate the fit of the item within the subscale and the total score. Item-total correlations of ≥0.5 and interitem correlations ≥0.3 were considered acceptable [45]. We hypothesized that individual items or indicators of the scale should all be measuring the same construct and thus be highly inter-correlated. The interclass correlation coefficients (ICC 2, 1; two observation time points of one item) was calculated in order to evaluate the reproducibility of the results (under constant condition). Single rating, absolute agreement, and a two-way mixed- effects model were used. We assumed that item scores of the two test results would be in agreement and ICC value ≥0.7 were satisfactory [42].

**Construct validity** was evaluated by factorial (exploratory and confirmatory factor analysis, discriminant and convergent validity) and known group validity (hypothesis testing) [46].

Exploratory factor analysis (EFA) is known as a data-driven method, and confirmatory factor analysis (CFA) as a theory-driven method. So the usage of EFA or CFA should be strictly considered and chosen according to the aim of a study, and aimless application of EFA and CFA to the same dataset should be avoided [47]. Latent variable structure of a dataset can be explored with EFA. On the other hand, CFA requires an a priori hypothesis or previous “theory” as CFA is a hypothesis testing method which tests whether the obtained dataset is suitable for a model [47]. Thus, first we used CFA to investigate whether the 9-factor structure can be replicated in the new dataset (model fit of the dataset obtained from 212 participants). CFA with maximum likelihood estimation was used for validation [48, 49]. The following goodness-of-fit indices were used to assess the model: Tucker Lewis Index (TLI; > 0.90 acceptable, > 0.95 excellent), the Comparative Fit Index (CFI; > 0.90 acceptable, > 0.95 excellent), and Root Mean Square Error of Approximation (RMSEA; < 0.08 acceptable, < 0.05 excellent), and Standardized Root Mean Residual (SRMR; < 0.08 acceptable) [50]. Second, after performing CFA, we extracted a more suitable factor structure from the same dataset. We then performed exploratory factor analysis (EFA) [48, 51]. Since our sample data violated the assumption of multivariate normality, EFA was performed using Principal axis factoring (PAF) extraction method [48, 49]. Extracted factors were rotated by oblique (promax) rotation [52]. Oblique rotation was chosen based on the expectation that dimensions of health would be associated [53]. Prior to conducting EFA, Bartlett’s test of sphericity (*p <* 0.05) [54] and the Kaiser–Meyer–Olkin (KMO *>* 0.5) measure of sampling adequacy [55] was performed to evaluate the factorability. The determination of the number of meaningful factors to be retained was guided by the scree plot test (above the break or elbow), Kaiser’s criteria (Eigenvalue≥1), interpretability, and the cumulative variance explained (> 40%) [56]. Items of the P-QoL were retained based on the following criteria: those with primary factor loadings > 0.4 and secondary factor loadings < 0.3 [51]. Items that did not meet these criteria were individually removed and the EFA repeated until all remaining items met these criteria for item retention. The reliability of items in each factors was examined using Cronbach’s alpha and value ≥0.7 for a factor was deemed reliable [30]. We also evaluated convergent and discriminant validity for the extracted factors. Factor-based convergent validity, the degree to which items within a single factor are highly correlated, was measured by composite reliability (CR ≥0.7) and average variance extracted (AVE ≥0.5) [57]. AVE < CR was used to establish convergent validity [58]. Factor-based discriminant validity, the extent to which factors are distinct and uncorrelated, was assessed by comparing AVE, maximum shared squared variance (MSV), average shared squared variance (ASV) and square root of AVE [59]. Discriminant validity was corroborated if AVE > MSV/ASV and the AVE square root of a given factor greater than inter-construct correlation [57]. Model validity measures was performed using “master Validity Tool”, AMOS Plugin [60].

Known group validity was evaluated by comparing the median-score distribution of P-QoL factors according to symptom status of participants. Women having POP symptoms are associated with poor HRQoL [18, 28]. Therefore, we tested the hypothesis that women with symptoms suggestive of POP would had a lower HRQoL scores as compared with those without symptoms of POP. The participants of this study were divided into two groups based on the symptom status (symptomatic vs. asymptomatic). Median P-QoL score of the two groups were tested using Mann-Whitney U test since our P-QoL score did not follow a normal distribution.

**Criterion validity**, how well the questionnaire correlates with an existing gold standard, was assessed by comparing P-QoL factors scores with the objective vaginal examination findings using SPOP-Q system [18]. Spearman’s correlation coefficient (SCC) was used to quantify the magnitude of the correlation. We used the following criteria to interpret the size of the correlation coefficients: 0.8–1.0 excellent, 0.61–0.80 very good, 0.41–0.60 good, 0.21–0.40 sufficient, and 0.00–0.20 poor [61]. We hypothesized that P-QoL score is correlated with SPOP-Q score and women with higher score of SPOP-Q had poor HRQoL.

We used the Analysis of Moment Structures (AMOS; version 23, Chicago, IL) for CFA, the Statistical Package for Social Sciences (SPSS; version 20, IBM Corp., Armonk, NY) for EFA, and STATA version 14 (StataCorp, College Station, TX, USA) for other calculations.

#### Ethical approval

Prior to study commencement, the developers’ authorization for the adaptation was obtained. Participation in the study was voluntary, and verbal and/or informed written consent was obtained prior to inclusion. The study was approved by the Institutional Review Committee at the University of Gondar (O/V/P/RCS/05/216/2017 on November 2017).

## Results

### Characteristics of participants

Of the 230 women invited to take part in the validation procedure, seven were excluded due to withdrawal before pelvic examination (*n* = 4) or were missing after completion of the interview (*n* = 3). In total, 223 women were enrolled in the final analysis, giving a response rate of 97%. Among these 223 participants, 152 (68.2%) were classified as symptomatic, and 71 (31.8%) as asymptomatic. The mean age and parity were 46.5 years (range 20 to 70) and 5.8 (range 0–12) respectively. Symptomatic women were older and had higher parity than asymptomatic women (*p* <  0.001). There were 139 symptomatic women (91.4%) in POP stage 3 or 4. However, 37 (52.1%) of asymptomatic women had no POP and none of them had more than stage 2. The characteristics of the study participants are presented in Table 1.

**Table 1:** Characteristics of study participants, University of Gondar, Ethiopia. (*n* = 223)

	Symptomatic *N* (%)	Asymptomatic *N* (%)	*P-value*
Age (years)			
Mean(+SD)	50.3 (11.3)	38.8 (13.6)	< 0.001^a^
Educational status*			
Illiterate	149 (98.0)	62 (87.2)	
Literate	3 (2.0)	9 (12.8)	
Parity			
Mean(+SD)	6.7 (2.7)	3.9 (3.3)	< 0.001^a^
POP-Q findings, *n* (%)			
Stage 0	0 (0)	37 (52.1)	
Stage 1	4 (2.6)	27 (38.0)	
Stage 2	9 (5.9)	7 (9.9)	
Stage 3	113 (74.3)	0 (0)	
Stage 4	26 (17.1)	0 (0)	
Total	152 (100%)	71 (100%)	

*SD* standard deviation, *POP-Q* Pelvic Organ Prolapse Quantification system

*Literate participants attended formal education at primary, secondary, preparatory or/and university/college level; illiterate participants did not attend formal education and include those who could and could not read and write

^a^Calculated with independent t-test

### Translation and adaptation of P-QoL scale

Stage 1- Forward translation was performed as planned without major difficulty. However, specific challenge related to the idiomatic usage of the word “prolapse” was found. It had several translation alternatives and required consideration by the committee of experts to reach a consensus to ensure semantic and idiomatic equivalence.

Stage 2- Principal investigator (TB) and other two bilingual experts prepared the synthesis version with the aid of both Amharic versions.

Stage 3- Backward translation was carried out as planned. Both backward translated versions were compared with the original and satisfactory similarity was noted. Since no changes were introduced by the original developer, this version was used for pre-testing.

Stage 4- This stage was also performed as planned and no major problem encountered. By considering the issues raised in the forward translation process (stage 1), panel of experts agreed to replace the “prolapse” with the meaning of “uterine prolapse”.

Stage 5- The following difficulties were encountered in pre-testing. Seven patient had difficulty in understanding the word ‘prolapse’. Hence, an optional word inside the bracket ‘a protrusion of womb or uterus’ was added to make this question comprehensible. The revised question read as ‘prolapse/protrusion of uterus/womb’ and asked to the same patients, and responded well to it. Due to cultural taboos prevalent in Ethiopia, women feel uncomfortable in talking about sexual behavior. Similar observation is noted while patients were asked to respond to questions found in PR domains (item 9 and 10). Patients initially felt hesitation in responding; however, on explaining, they could realize the importance of such questions and answered appropriately.

Comments were analyzed by the committee of experts. After judging the comments made by participants during the pre-test, and resolved by consensus, the committee of experts drafted the final translated version of P-QoL and adopted for use in the psychometric evaluation. The final Amharic version of the P-QoL questionnaire is shown in the “Additional file 1”.

### Acceptability

All participants responded to all items in the Amharic P-QoL questionnaire, and marked legibly and correctly (no missing items found). Data collectors reported no difficulties in asking the items and no patients reported having met problems in understanding the items. The average time taken to complete the questionnaire was 6 min.

### Content validity

Content validity was considered adequate according to the criteria and the arguments made by the committee of experts during the process of adaptation and the qualitative analysis of participant/women comments. All of them agreed on all of the proposed translated items as acceptable. The average scale content validity (CVI) was 0.98, which is above the cut-off of 0.80. No changes, including relevant items that need to be added, were made to the items as result of the content validity review. Participant interviewees reported that in general the items in the questionnaire were clear and comprehensible achieving face validity. However, they suggested a few changes when drafting a final version of the instrument.

### Evaluation of psychometric properties

#### Reliability and item analysis

Internal consistency of the translated version was 0.96 [95% confidence interval (CI)] 0.95–0.97; *p* <  0.001). The average interitem correlation was 0.55 with the individual correlations ranging from 0.23 to 0.88, suggesting good reliability. The average item- total correlation was 0.68. The correlations between the 20 items of the P-QoL and the total scores ranged from 0.49 to 0.88, indicating good relationship between each item and all the other items on the scale. As seen in Table 2, the magnitude of change in Cronbach’s alpha was almost uniform across items, and in no instances did removal of an item from the scale result in an increase in the value of Cronbach’s alpha.

**Table 2:** Internal consistency, item-total correlations, and alpha if item deleted from the Amharic Pelvic Organ Prolapse Quality of Life (P-QoL) questionnaire

Item	Item description	Corrected item-total correlation	Alpha if item deleted
1	Present general health	0.65	0.96
2	Prolapse impact on life	0.79	0.95
3	Impact on Household or role	0.83	0.95
4	Impact on activities outside the home	0.80	0.95
5	Impact on physical activities	0.84	0.96
6	Impact on travel ability	0.86	0.96
7	Limit social life/activities/work	0.88	0.94
8	Limit ability to visit friends/families	0.85	0.94
9	Affect relationship with partner/spouse	0.71	0.95
10	Affect sexual life with spouse	0.66	0.96
11	Affect family life	0.60	0.96
12	Feel depressed	0.78	0.95
13	Feel anxious/nervous	0.71	0.95
14	Feel bad about yourself	0.64	0.96
15	Affect sleep/bad dreams	0.60	0.96
16	Feel worn-out/tired	0.62	0.96
17	Use pad to protect	0.49	0.96
18	Manually push up	0.69	0.95
19	Pain/discomfort	0.73	0.95
20	Prevent standing	0.77	0.95
	Overall Cronbach’s alpha (20 items)		0.96
	Standardized item alpha		0.96

Ten women reported a change in POP severity and were removed from the test-retest analysis. The second test was performed with a median of 12 days (range 8–21 days) after baseline. The result revealed excellent test-retest reliability between the paired scores for all the domains (ICC = 0.87 [95% confidence interval (CI)] 0.82–0.92; *p* < 0.001).

#### Confirmatory factor analysis

The hypothesized 9-factor model had inadequate fitness indicators (CFI = 0.69, RMSEA = 0.17, SRMR = 0.43, and TLI = 0.65).

#### Exploratory factor analysis

The KMO measure of sampling adequacy was 0.95 and the significance of Bartlett’s test of sphericity was less than 0.001 (χ^2^ = 4086.55, *df* = 190), meaning that EFA can be applied to the obtained dataset. A total of three factors were extracted and rotated, and the cumulative variance explained was 70.01%. Factor loadings indicate that individual item reliability was adequate for all items, ranging from 0.50 to 0.95 for the three factor model. Twelve items, i.e., from #1 to #8, and #17 to #20, were entered with item loadings ranging from 0.47 to 1.03 in Factor 1, which was designated physical function. Five items (#12, #13, #14, #15, and #16) were entered for Factor 2, which was designated psychological function. Three items (#9, #10, and #11) were entered for Factor 3, which was designated social/personal relationship. Cronbach alpha was greater than 0.85 in all factors. Average variance extracted and Composite reliability showed adequate values. The result demonstrated AVE >  0.5, AVE < CR and CR >  0.7 in all factors, indicating convergent validity of the extracted construct. Similarly, both extracted factors were distinct showing divergent validity. AVE was greater than MSV and ASV, and square root AVE was greater than inter-construct correlation. EFA with factor loading of items is shown in Table 3.

**Table 3:** Exploratory factor analysis with factor loadings for the Amharic Pelvic Organ Prolapse Quality of Life (P-QoL) questionnaire

Items	Item description	Factor 1	Factor 2	Factor 3
Item 1	Present general health (GHP)	**0.56**	0.12	0.02
Item 2	Prolapse impact on life (PI)	**0.85**	0.00	0.02
Item 3	Impact on Household or role (RL1)	**1.03**	−0.11	− 0.01
Item 4	Impact on activities outside the home (RL2)	**1.03**	−0.13	−0.03
Item 5	Impact on physical activities (PL1)	**0.84**	0.04	0.06
Item 6	Impact on travel ability (PL2)	**0.84**	0.11	−0.01
Item 7	Limit social life/activities/work (SL1)	**0.78**	0.15	0.05
Item 8	Limit ability to visit friends/families (SL2)	**0.69**	0.12	0.15
Item 9	Affect relationship with partner/spouse (PR1)	0.07	0.03	**0.81**
Item 10	Affect sexual life with spouse (PR1)	−0.09	−0.03	**1.02**
Item 11	Affect family life (PR3)	0.17	0.07	**0.49**
Item 12	Feel depressed (E1)	0.14	**0.74**	0.07
Item 13	Feel anxious/nervous (E2)	−0.05	**0.99**	−0.05
Item 14	Feel bad about yourself (E3)	−0.06	**0.78**	0.07
Item 15	Affect sleep/bad dreams (SE1)	0.27	**0.64**	−0.04
Item 16	Feel worn-out/tired (SE2)	0.03	**0.44**	0.00
Item 17	Use pad to protect (SM1)	**0.49**	0.09	−0.06
Item 18	Manually push up (SM2)	**0.59**	0.12	0.03
Item 19	Pain/discomfort (SM3)	**0.47**	0.34	0.00
Item 20	Prevent standing (SM4)	**0.52**	0.30	0.04
	Cronbach’ alpha	0.96	0.87	0.86
	Average variance extracted (AVE)	0.56	0.55	0.65
	Composite reliability (CR)	0.92	0.85	0.84
	Maximum shared squared variance (MSV)	0.50	0.50	0.45
	Average shared squared variance (ASV)	0.48	0.44	0.41
	Square root of AVE (√AVE)	0.74	0.74	0.80

Items with high loading (> 0.4) to factors are indicated in bold

Extraction Method: Principal Axis Factoring

Rotation Method: Promax with Kaiser Normalization

#### Known group validity

There were statistically significant differences among the two groups in the P-QoL scores (Mann-Whitney U test; *p* < 0.001). The median P-QoL domain scores were higher in symptomatic women compared to asymptomatic showing a worse HRQoL in the former group (Table 4).

**Table 4:** Comparison of the Amharic Pelvic Organ Prolapse Quality of Life (P-QoL) domain scores between symptomatic and asymptomatic women

Factors extracted (P-QoL Domains)	Symptomatic Median (IQR)	Asymptomatic Median (IQR)	Mann –Whitney U test	*P-value* ^*a*^
Physical function (Factor 1)	75 (83, 64)	36 (52, 22)	1423	< 0.0001
Psychological function (Factor 2)	67 (80, 53)	40 (60,13)	2857	< 0.0001
Social/personal relationship (Factor 3)	67 (78,44)	22 (44,0)	2212	< 0.0001

^a^ Calculated using the Mann–Whitney test

Interquartile ranges (IQR)

#### Criterion validity

Spearman’s correlation coefficients between extracted P-QoL domain score and the SPOP-Q scores were from 0.42 and 0.64, indicating a low to moderate strength of association. Both correlation coefficients were significant at *p* < 0.001(Table 5).

**Table 5:** Spearman’s correlation coefficient (SCC) between the Amharic Pelvic Organ Prolapse Quality of Life (P-QoL) domain scores and vaginal examination (SPOP-Q stage) in symptomatic women

P-QoL Domains	SCC	P-value*
Physical function (Factor 1)	0.64	< 0.0001
Psychological function (Factor 2)	0.42	< 0.0001
Social/personal relationship (Factor 3)	0.51	< 0.0001

*Calculated using Spearman’s rank correlation (SCC) analysis

## Discussion

### Summary of main findings

Like other diagnostic procedures, HRQoL measures should be valid, reliable, and sensitive over time [62]. P-QoL has proven to be valid and reliable instrument for assessment and management of women with POP symptoms in clinical and research practice [6, 14, 15]. Until now, their Amharic translation has never been validated. In the present study P-QoL questionnaire was translated in Amharic and its reliability and validity were assessed.

The P-QoL scale was successfully translated and culturally adapted to Amharic. The pilot study showed that it worked well, although some minor changes had to be made in finalizing the local language version to increase its technical equivalence. The Amharic version demonstrated excellent reliability and construct validity. Internal consistency was very high and satisfactory agreement was observed between the paired test-retest scores. With regard to the overall score, ICC values were between 0.82 and 0.92 which is indicative of very good to excellent agreement.

Based on CFA indices, this sample has unacceptable fit to the 9-factor model. EFA found a 3-factor structure model from the dataset. In EFA, communalities and factor loadings for all the items were well above the cutoff values. All extracted factors showed good discriminant and convergent validity. We observed good correlations between P-QoL and SPOP-Q scores. The Amharic version was capable of detecting the difference of P-QoL score between symptomatic and asymptomatic groups.

### Translation and adaptation

The cross-cultural adaptation was performed using a systematic approach [33], including different steps. Both forward-and-backward translations were performed as planned and there were no changes in the instruction, and lay-out of the questionnaire. But selected items proved difficult to translate and were changed. All changes had the purpose to optimize the comprehensibility of the questionnaire and were discussed with the members of the research team. Although difficulties were encountered during pre-testing, especially in understanding some terminology, the result suggest the Amharic version of the P-QoL has good acceptability. The absence of difficulty in responding the majority of items, the ease of completion within short period of time, and appropriately responding to those difficult but revised questions provides evidence for the acceptability of the instrument.

Content validity was determined in a similar way as described by previous validation studies of the P-QoL [28, 29]. Emphasis was given to maintain the original context and meaning of the words rather than a direct word by word translation [42]. We found the Amharic questionnaire as content valid after excellent expert panel agreement on the relevance of items [36] and reviewed by multilingual expert translators. Moreover, it appears acceptable to patients and does not constitute an extra burden to the professionals using it. We concur with other investigators that the P-QoL is easy to use in a busy clinical setting [18, 22].

### Reliability

In this study, the Amharic version of P-QoL demonstrated excellent internal consistency (0.96). This finding was comparable to other studies which demonstrated a Cronbach’s alpha scores of between 0.84 and 0.93 [18, 24, 27, 63] and considerably higher than the traditional threshold of 0.7 [30], indicating inter correlation of the items found in the instrument. Average interitem correlation and average item-total correlation were also high, suggesting good reliability of the instrument. These high internal reliability of the instrument may be sufficient for individual clinical use as well as use for research groups, according to Bland and Altman [64].

The 2-week test–retest reliability result also demonstrated excellent correlation between paired test–retest scores (ICC for agreement 0.87; *p* < 0.001). The duration was chosen because it is long enough to avoid recall bias and short enough for the condition to stay unchanged [18, 42]. The result is comparable with the English (0.64–0.83) [18], Persian (0.76–0.95) [27] and Dutch (0.89–0.99) [23] validation studies, ensuring that responses are not too varied across time periods. So measurement taken at any point in time using the Amharic P-QoL is reliable. This may encourage researchers in the future to interpret their results from the Amharic version.

### Validity

In this study, CFA result showed unacceptable model fit to the 9-factor model. Also, we conducted EFA to extract the new factor structure of the dataset and found a three-factor structure model. However, this factor structure is inconsistent with the factor structure reported in the original English version [18] and other validation studies [25, 27, 28]. But as to the construct validity, current study strongly supported the multidimensionality of the scale and corroborate with the existing literature [18, 23, 24]. All of the extracted factors showed acceptable Cronbach alpha and demonstrated good convergent and divergent validity. Moreover, median P-QoL domain scores were significantly higher in the symptomatic women compared to asymptomatic women (known group validity). And this pattern of P-QoL scores observed among groups suggests that the questionnaire is useful for assessing HRQoL in women with POP symptoms. The good discrimination ability of the Amharic measure among groups supports its high construct validity.

The correlation between the Amharic P-QoL score with SPOP-Q stage were calculated for criterion (concurrent) validity. Although there is no gold standard to determine HRQoL in women with POP [42], SPOP-Q score was taken as a reference standard [18, 28, 64] and evidence showed a strong correlation between P-QoL and POP-Q stage [18, 27, 65]. In this study we observed good correlation between P-QoL and SPOP-Q scores (*p* < 0.001), indicating a higher stage associated significantly with worse P-QoL scores, especially in women with symptom suggestive of POP. The correlation between scores and vaginal finding in other studies [18, 23, 27] was almost similar to our study, though they differ in strength of correlation. Exhibiting correlation with the stage of POP doesn’t mean P-QoL assessment substitute or replace physical examination.

Strengths of this study are the adoption of a multistep translation method, as supported by existing evidence rather than the simple translation/back-translation process [33, 34], and used COSMIN recommendations for reporting of measurement properties [46], which is the current reference standard for reporting measurement properties as proposed by Terwee et al. [30]. Specific limitations, however, must considered when interpreting this findings. First, our study was conducted in a single urban hospital; therefore, results may not be generalizable to populations in rural and remote areas. Specifically, rates of illiteracy may impact validity. Further validation studies in more general contexts are therefore recommended. Second, responsiveness to change and minimal clinically important difference (MCID) were not evaluated because of logistics problems. Since these are an important scale property to determine the utility of the Amharic P-QoL scales as outcome measures, we recommend inclusion of this in future studies. Third, since there were no validated questionnaires in the Amharic language, we failed to use other criterion comparators for both P-QoL item and domain values. We used the SPOP-Q stage as a gold standard criterion. Fourth, sensitivity of the topic being studied carries the risk of providing socially desirable answers instead of true responses.

## Conclusions

The P-QoL was successfully translated and culturally adapted into Amharic. The Amharic version achieved good conceptual and content equivalence. The translated version was valid and reliable measure to assess POP symptom severity and its impact on HRQoL in Amharic-speaking Ethiopian women at the outpatient health care setting. The questionnaire is easily understandable, and can be administered and completed by patients and used in clinical practice. Further studies are needed to evaluate responsiveness of P-QoL.

## Additional file

Additional file 1: Amharic version of P-QoL questionnaire. (DOCX 481 kb)

## Abbreviations

CFA: Confirmatory Factor Analysis
COSMIN: Consensus-based Standards for the Selection of Health Measurement Instruments
CVI: Content Validity Index
EFA: Exploratory Factor Analysis
HRQoL: Health Related Quality of Life
ICC: Intraclass Correlation Coefficient
MCID: Minimal Clinically Important Difference
PGIC: Patients’ Global Impression of Change
POP: Pelvic Organ Prolapse
P-QoL: Prolapse Quality of Life
PROM: Patient Reported Outcome Measures
SCC: Spearman Correlation Coefficient
SPOP-Q: Simplified POP-Quantification
