# Supplementary figures and images for: Translation, transcultural adaptation, reliability and validation of the pelvic organ prolapse quality of life (P-QoL) in Amharic
Source: Health Qual Life Outcomes. 2019 Jan 14;17:12. doi: 10.1186/s12955-019-1079-z (PMC6332683; doi:10.1186/s12955-019-1079-z)

**Additional file 1: Amharic version of the P-QoL questionnaire**


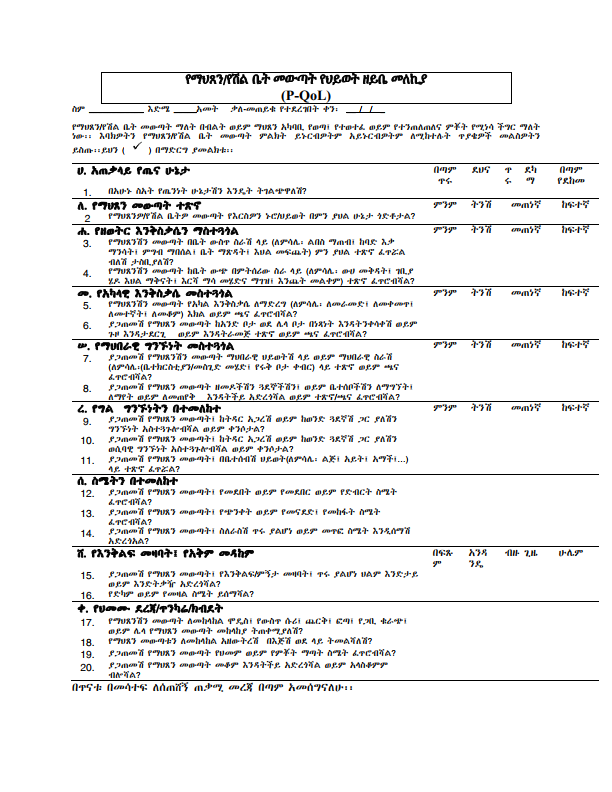

Supplement: Supplementary file 1 — Amharic version of P-QoL questionnaire. (DOCX 481 kb) [file 12955_2019_1079_MOESM1_ESM.docx]
